# CoCMA: Energy-Efficient Coverage Control in Cluster-Based Wireless Sensor Networks Using a Memetic Algorithm

**DOI:** 10.3390/s90604918

**Published:** 2009-06-22

**Authors:** Joe-Air Jiang, Chia-Pang Chen, Cheng-Long Chuang, Tzu-Shiang Lin, Chwan-Lu Tseng, En-Cheng Yang, Yung-Chung Wang

**Affiliations:** 1 Department of Bio-Industrial Mechatronics Engineering, National Taiwan University, Taipei 106, Taiwan; E-Mails: supercjb@pie.com.tw (C.C.); clchuang@ieee.org (C.C.); r96631025@ntu.edu.tw (T.L.); 2 Department of Electrical Engineering, National Taipei University of Technology, Taipei 106, Taiwan; E-Mails: cltseng@ee.ntut.edu.tw (C.T.); ycwang@ee.ntut.edu.tw (Y.W.); 3 Department of Entomology, National Taiwan University, Taipei 106, Taiwan; E-Mail: ecyang@ntu.edu.tw (E.Y.)

**Keywords:** wireless sensor network, sensing coverage, energy efficiency, memetic algorithm

## Abstract

Deployment of wireless sensor networks (WSNs) has drawn much attention in recent years. Given the limited energy for sensor nodes, it is critical to implement WSNs with energy efficiency designs. Sensing coverage in networks, on the other hand, may degrade gradually over time after WSNs are activated. For mission-critical applications, therefore, energy-efficient coverage control should be taken into consideration to support the quality of service (QoS) of WSNs. Usually, coverage-controlling strategies present some challenging problems: (1) resolving the conflicts while determining which nodes should be turned off to conserve energy; (2) designing an optimal wake-up scheme that avoids awakening more nodes than necessary. In this paper, we implement an energy-efficient coverage control in cluster-based WSNs using a Memetic Algorithm (MA)-based approach, entitled CoCMA, to resolve the challenging problems. The CoCMA contains two optimization strategies: a MA-based schedule for sensor nodes and a wake-up scheme, which are responsible to prolong the network lifetime while maintaining coverage preservation. The MA-based schedule is applied to a given WSN to avoid unnecessary energy consumption caused by the redundant nodes. During the network operation, the wake-up scheme awakens sleeping sensor nodes to recover coverage hole caused by dead nodes. The performance evaluation of the proposed CoCMA was conducted on a cluster-based WSN (CWSN) under either a random or a uniform deployment of sensor nodes. Simulation results show that the performance yielded by the combination of MA and wake-up scheme is better than that in some existing approaches. Furthermore, CoCMA is able to activate fewer sensor nodes to monitor the required sensing area.

## 1. Introduction

A wireless sensor network (WSN) is a telecommunication network that consists of a number of wireless sensor nodes. Given the limited energy and communication range of wireless sensor nodes, many previous studies have attempted to improve the quality of service (QoS) through prolonging network lifetime. Some fundamental issues have also been investigated to enhance the reliability of WSNs, such as power management [1], routing protocols [2], localization [3], medium access control (MAC) [4], and coverage control [5,6]. For mission-critical applications for WSNs, such as military surveillance [7] and object tracking [8], full sensing coverage is crucial for event detection. As a result, how well the deployed sensor nodes cover a target area is a fundamental criterion to evaluate QoS. Usually, both minimizing the energy consumption and extending the lifetime are also essential.

In general, the *k*-coverage problem has been discussed when coverage control for WSNs is considered [5,6]. The *k*-coverage means that each point is within the sensing range of *k* or more sensor nodes. The value of *k* would be determined in different application-specific WSNs. For example, in [9] the authors consider the *m*-coverage problem in a target tracking application, where every target should be monitored by *m* sensor nodes. They then optimize the accuracy of target tracking and power consumption using the genetic algorithm (GA). In this paper, we consider 1-coverage problem for a WSN, where each point of interest (POI) is covered by one sensor node at least. Usually, in order to maintain a maximum sensing coverage area with the lowest power consumption, the redundant sensor nodes chosen by node-scheduling algorithms [10-12] should enter an inactive mode (sleeping mode) to reserve battery power. If one active node runs out of its energy, one or more of the inactive nodes needs to be awakened instantly to replace the dying node. These node-scheduling algorithms guarantee 100% sensing coverage as long as possible. In addition, the approach in [13] adjusts the sensing ranges of nodes dynamically to improve the sensing coverage. They utilized density control and various patterns of node placements to evaluate the performance of coverage preservation.

On the other hand, the problem of selecting a minimum number of sensor nodes to cover all the required POIs belongs to the NP-Complete problem [14], also named the set covering problem (SCP) [14-16]. Some studies have applied optimization algorithms to the SCP. Jia *et al.* presented an elitist non-dominated sorting genetic algorithm (NSGA-II) to find the redundant nodes and turn them off in order to conserve energy [17]. Lin *et al.* employed the ant colony algorithm to solve the SCP [14]. Since the evaluation of area coverage is complicated, some studies used point coverage to approximate area coverage [18]. In this paper, we consider a SCP with a number of POIs deployed in a sensing field. In coverage control, we concern whether each POI in a sensing field that can be monitored by at least one sensor node at different time (i.e., the 1-coverage problem). Thus, the coverage control should guarantee that the original coverage is maintained after turning off redundant nodes.

Memetic algorithms (MAs) are population-based heuristic search approaches for optimization problems [19]. MAs are similar to GAs, but MAs incorporate local search with GAs. Inspired by the notion of meme presented by Dawkins [20], MAs employ one or more problem-specific heuristic searching to improve the solutions generated by GA operators, such as crossover and mutation. Hence, the performances of MAs are generally better than those of GA. Particularly, MA is a very suitable optimization algorithm for complex problems, such as the traveling salesman problem [21], the graph bi-partitioning problem [22], and binary quadratic programming [23]. MA has also been utilized to solve the minimum energy network connectivity (MENC) in WSNs [24]. The MENC problem is to simultaneously minimize the power consumption on each node and to maintain the global connectivity of the network, which belongs to the NP-Complete problem. Additionally, the optimal operation mode for each node that can be a cluster head or a regular sensor node with a high or low transmitting range of signals is determined by the MA, so that the energy consumption can be minimized [25]. The energy consumption for each operation mode is different. These two [24,25] do not consider sensing coverage requirements when using MAs to optimize sensor networks. In this paper, we utilize the MA to optimize the sensing coverage of a CWSN while minimizing the number of activated nodes (i.e., the SCP encountered in a given CWSN).

This study presents a MA-based coverage-preserving algorithm to optimize the point coverage of a CWSN. We investigate how the MA can be applied to an optimization problem in CWSNs. Note that many sensor nodes may be redundant after all of the nodes are deployed. In general, redundant sensor nodes cover sensing areas that are overlapped with other neighboring sensor nodes. In order to reduce energy consumption, the CoCMA determines which sensor nodes should be switched to sleeping modes. The CoCMA can also awaken some of the sensor nodes when sensing coverage declines. This step is called the wake-up scheme, which is different from that in the MA. Using evolutionary operations, our CoCMA is time consuming, so we develop a wake-up scheme that is less complex to determine an optimal schedule for nodes, awakening one or more sensor nodes near a dying one. The lost coverage of POIs can therefore be retrieved. The CoCMA can allow a minimum number of active sensor nodes to achieve maximum coverage and prolong the network lifetime simultaneously. This is very different from the aforementioned algorithms; for example, the NSGA-II [17] only considers coverage optimization in the phase of the initial deployment without considering long-term operation. The CoCMA can be incorporated into different routing protocols. We have applied the proposed CoCMA to a CWSN [26] and compared their performances with the existing approaches in [26-29]. Simulation results show that the proposed CoCMA can not only prolong the network lifetime considerably but also maintain the higher sensing coverage.

The remainder of this paper is organized as follows. Section 2 describes the preliminary models and assumptions. In Section 3, we formulate the SCP and present the energy-efficient coverage control that employs a MA to solve the SCP. Section 4 shows our experimental results in comparison to different approaches and then provides discussions on the comparisons. Finally, Section 5 gives our conclusions.

## 2. Preliminaries

In this paper, we assume that all of the wireless sensor nodes (referred to sensor nodes or nodes) and the sink node (sink) are stationary after deployment. These nodes are assumed to be homogenous, with the same communication capacity, sensing range, and data processing capacity. In addition, we assume that the position of every sensor node is known *a priori*, and the sink is aware of the positions of all sensor nodes in this study. The sensing area for a sensor node is assumed a disk-shaped area with a specific diameter (*r_s_*). In fact, the CoCMA can be applied to WSNs with irregular-shaped sensing areas as long as their sensing coverage models are given.

**Figure 1.**
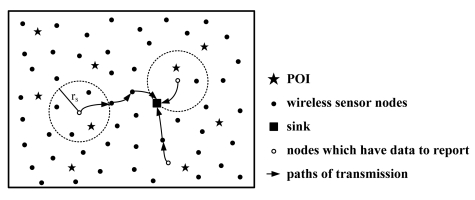
For the coverage-preserving application for a WSN, the events at the position marked by stars will be detected and reported to the sink node through the surrounding nodes.

The proposed approach considers the point coverage problem in a given area. The POI is the place where the event occurs. We assume that an event signal is always generated at each POI. As shown in Figure 1, each POI is surrounded by a number of sensor nodes. If the POI is located within the sensing range *r_s_* of a given sensor node, the sensor node will detect the event signal and transmit the measured data to the sink by a multi-hop manner.

### 2.1. Sensing Coverage Model

*A* given target area *R* is a two-dimensional plane of *L_x_ × L_y_* m^2^. A set of sensor nodes in *R* is thus defined as *C* = {*c*_1_, *c*_2_, *c*_3_, …, *c_N_*}, where *c_i_* = {*x_i_, y_i_, r_s_*}, *i*∈[1, *N*] and *N* is the total number of deployed sensor nodes. The coordinate and sensing radius of a sensor node are {*x_i_, y_i_*} and *r_s_*, respectively. A set of POIs distributed over the region *R* is defined as *P* = {*p*_1_, *p*_2_, *…, p_M_*}, where *p_j_* is a POI located at {*x_j_, y_j_*}, *j*∈[1, *M*], *and M* is the number of POIs. In addition, we introduce a binary variable ℜ*_i,j_* to represent whether *c_i_* is able to cover *p_j_. ℜ_i,,j_* is defined as:

(1) $${ℜ}_{i,j}=\{\begin{array}{ll} 1 & \text{if}{\left(x_{i}-x_{j}\right)}^{2}+{\left(y_{i}-y_{j}\right)}^{2}<r_{s}^{2}\\ 0 & \text{otherwise}. \end{array}$$

If the distance between *p_j_* and *c_i_* is less than *r_s_*, then *p_j_* is covered by *c_i_* (i.e., ℜ*_i_,_j_* = 1). Thus, the event signal generated at the POI can be detected. However, *p_j_* may be covered by *v* sensor nodes at the same time. For a particular *p_j_*, if it is covered by two or more sensor nodes (i.e., *c*_1_, *c*_2_, …, and *c_v_, v* ≤ *N*), the union of ℜ_1_, *_j_*, ℜ_2_,*_j_*, …, and ℜ*_v_*, *_j_* for *p_j_* can be calculated using the basic operation of the Boolean algebra:

(2) $${ℜ}_{1,j}\vee {ℜ}_{2,j\ldots }\vee {ℜ}_{v,j}=1-{\bar{ℜ}}_{1,j}\wedge {\bar{ℜ}}_{2,j\ldots }\wedge {\bar{ℜ}}_{v,j},$$

where ℜ̂*_i,j_* is the Boolean inverse of ℜ*_i_,_j_*, i.e., ℜ̂*_i_,_j_* = 1 *−*ℜ*_i,j_*, “v” denotes the Boolean union, and “∧ ” denotes the Boolean intersect.

### 2.2. Energy Consumption Model

**Figure 2.**
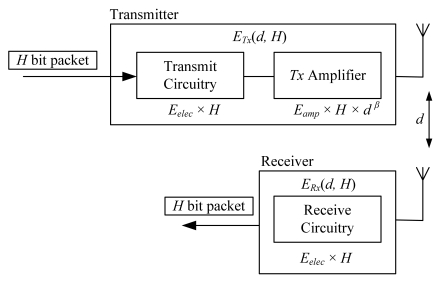
The energy consumption model used in this study.

According to a previous study [26], the cluster-based WSN (CWSN) should be an optimal design so that energy dissipation over the entire network is equally distributed, and the lifetime of the network can be prolonged. The CoCMA was designed to follow this suggestion. In order to compare the performance of the proposed algorithm with existing ones, we use the model in the previous study [26] to measure the energy consumption for both transmission and reception. This energy consumption model is depicted in Figure 2. Given that energy consumption in radio transmission is often greater than that in general sensor operations or memory access [30,31], we consider the energy consumption for radio transmission and ignore energy consumption for other operations.

In Figure 2, *E_elec_*denotes the dissipated energy for the transmit circuitry or receive circuitry per bit (in nano-Joules), *E_amp_* denotes the energy consumption for the power amplifier per bit, and *β* denotes the exponent for path loss. Consequently, when a transmitter sends an *H*-bit packet to a receiver, the total energy consumption can be calculated by:

(3) $$\begin{array}{l} E_{Tx}(d,H)=E_{\text{elec}}\times H+E_{\text{amp}}\times H\times d^{\beta }\\ E_{Rx}(d,H)=E_{\text{elec}}\times H, \end{array}$$

where *E_Tx_* and *E_Rx_* are the total energy consumption on transmitting and receiving an *H*-bit packet, respectively. Since all of the sensor nodes are assumed homogeneous, every sensor node has the same initial energy. The energy-rich sink, however, does not involve this energy consumption model; rather, it presumably comes from other power sources with plenty of supply.

## 3. Coverage Control Using Memetic Algorithm

### 3.1. The Set Covering Problem (SCP)

The SCP, one of the NP-Complete problems, was first defined in [14]. We apply this definition to the node scheduling optimization here. In this study, the SCP involves the issue of managing coverage with energy efficiency. SCP is to find a set of nodes with the minimum cost so that each POI is covered by at least one node. Thus, the SCP considered in the CoCMA can be formulated mathematically as follows:

(4) $$\begin{array}{l} \text{Optimization Model}:\\ \begin{array}{cc} \min\sum_{i}g_{i}\cdot x_{i} & i\in [1,N] \end{array} \end{array}$$

(5) $$\begin{array}{l} \text{Subject to}:\\ \begin{array}{cc} \sum_{i}{ℜ}_{i,j}\cdot x_{i}\geq 1, & j\in [1,M] \end{array}\\ \begin{array}{cc} x_{i}=1\;\text{or}\;0, & i\in [1,N], \end{array} \end{array}$$

where *g_i_* is the cost of activating each node; *x_i_*’s are the key decision variables that need to be determined by the CoCMA (e.g. one is for activation, and zero is for inactivation). The objective function in Equation (4) minimizes the total number of nodes needed to be activated. In this paper, we suppose that the cost of activating each node is identical (i.e., *g_i_* = 1). The constraint described in Equation (5) stipulates that every *p_j_* must be covered by at least one node. When the network is activated, using the optimization model depicted in Equations (4) and (5) is critical, since the model improves energy efficiency of the network.

Meta-heuristics are a group of approximate methods, designed to cope with optimization problems. For example, genetic algorithms, memetic algorithm (MA), problem-space search, neural networks and simulated annealing are all members of the family of meta-heuristics. In order to efficiently find the optimal solutions, we develop a meta-heuristic method that employs an iterative generation process to explore the search spaces based on learning strategies [32]. Based on these reasons, we propose the CoCMA to solve the SCP using a MA and an optimization strategy, named the wake-up scheme. The proposed CoCMA will be described in detail as follows.

### 3.2. The CoCMA Proposed for Cluster-based WSNs

**Figure 3.**
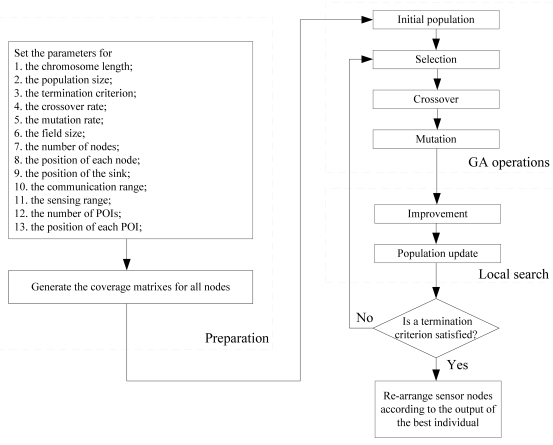
Flowchart of the optimization strategy using MA in the CoCMA.

In this study, we propose a method, called Coverage Control Using Memetic Algorithm (CoCMA), and apply it to CWSNs. The CoCMA contains two optimization strategies: a MA-based schedule for sensor nodes and a wake-up scheme. The first optimization strategy inactivates the redundant sensor nodes in a given CWSN according to the MA-based schedule for sensor nodes. The flowchart of the MA procedure is illustrated in Figure 3. In addition, the wake-up scheme is required to handle the energy-efficient coverage optimization in each round. In CWSNs, the word “round” represents a basic time unit. As mentioned above, MA consists of two parts, GA operations and local search. The initial population is usually generated randomly or by a specific manner. The evolutionary process is carried out in the GA operations, including selection, crossover and mutation.

In the CoCMA, every individual represents a solution with a corresponding fitness value, derived from a fitness function. The fitness function is used to represent the goodness of a solution. After completing the GA operations, the local search is utilized to further enhance the goodness of the solutions. By doing so, a new population of superior individuals is generated, and, the individuals in the new population are much closer to the global optimal solution. In addition, the evolutionary process will be terminated (i.e., the optimal solution is found) if a termination criterion is satisfied. In following subsections, the CoCMA will be presented in detail.

#### 3.2.1. Genetic Representation

Designing a genetic representation that focuses on a given problem is essential for MAs and GAs. A better genetic representation can improve the performance of evolution. For the energy-efficient coverage optimization, it is necessary to convert the schedule for nodes to genetic representations. In this study, an optimal schedule for nodes is used to activate or inactivate sensor nodes in CWSNs at a specific time, so, the network lifetime can be prolonged. For the schedule, solutions are represented by binary strings of 0s and 1s, and the status of a node is represented by an allele. For example, 0 denotes that the sensor node is inactivated, while 1 denotes that the node is activated.

**Figure 4.**
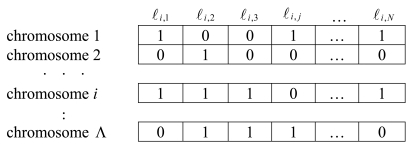
Genetic representation used in the CoCMA.

**Figure 5.**
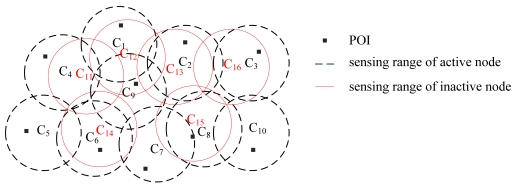
Illustration of sensing coverage in a CWSN for environmental sensing application. In this case, 16 nodes are activated to cover 10 POIs.

The genetic representation for the energy-efficient coverage optimization is depicted in Figure 4. In this figure, Λ denotes the number of the individuals (chromosomes), and allele ℓ*_i_,_j_* denotes the status of sensor node *cj* in chromosome *i*. Note that the length of each chromosome is *N*. Since various population sizes lead to different population diversities, it is critical for the CoCMA to use an appropriate population size. According to the review paper presented by Eiben *et al* [33], the optimal population size can be controlled by an innovative way [34]. In their discussion, the population size of GA can be one of the variables. Thus, the resulting system favors the propagation of better genes. In order to simplify this work, the population size of CoCMA is set depending on the general experience before activating the CoCMA. Therefore, the population size is fixed for every population generated in each generation. As depicted in Figure 5, there are sixteen nodes used for environmental sensing, and some redundant nodes (red circle) are found. In order to save energy and achieve the optimal coverage ratio, an optimal schedule for nodes is integrated into the CoCMA to inactive these redundant nodes. From the viewpoint of the genetic representation; we use a string “1111111111000000” to express a chromosome in the example given in Figure 5.

#### 3.2.2. Fitness Function

The fitness function is a measure that indicates the goodness of a particular chromosome. The objective of the CoCMA is to achieve the optimal coverage ratio using fewer sensor nodes. As a result, the goodness of schedule for active sensor nodes is regarded as the fitness function of the proposed CoCMA.

For each node, a coverage vector is proposed to represent the coverage of POIs. According to the sensing coverage model described in Section 2.1, the coverage vector of one particular node *ci* can be defined as *π_i_ =*[ℜ*_i_*,_1_, ℜ*_i_*_, 2_, *…*,ℜ*_j_,_M_*], where *c_i_* ∈*C*. Likewise, for another sensor node *c_j_*∈*C*, the coverage vector is denoted by *π_j_ =* [ℜ*_j_*_,1_, ℜ*_j_*_,2_, …, ℜ*_j,M_*], where *i≠j*. We use a binary model to represent whether a given POI is covered by a sensor node. The Boolean operations is applied to the vector. A synthetic coverage vector of *c_i_* and *c_j_* can be then calculated by performing the Boolean union between *π* and *π_j_*:

(6) $$\begin{array}{l} \varpi (c_{i},c_{j})=\pi_{i}\vee \pi_{j}\\ \;=[{ℜ}_{i,1}\vee {ℜ}_{j,1},{ℜ}_{i,2}\vee {ℜ}_{j,2},\ldots ,{ℜ}_{i,M}\vee {ℜ}_{j,M}], \end{array}$$

where *ϖ* denotes a synthetic coverage vector, which determines whether every POI is covered by *c_i_, c_j_* or not covered. Therefore, the synthetic coverage vector for a particular chromosome *k* is defined as follows:

(7) $$\varpi (k)=(l_{k,1}\cdot \pi_{1})\vee (l_{k,2}\cdot \pi_{2})\ldots \vee (l_{k,N}\cdot \pi_{N}).$$

Since the coverage evaluation process is simplified into binary operations, the performance of the CoCMA can be improved greatly. In addition, we can calculate the coverage ratio for the chromosome *k* by:

(8) $${ɛ}^{k}=\frac{{\left\|\varpi (k)\right\|}^{2}}{M},$$

where ‖*ϖ*(*k*)‖^2^ represents the number of active nodes. The utility ratio ${ℵ}_{t}^{k}$ of nodes for chromosome *k* is calculated by:

(9) $${ℵ}_{t}^{k}=\frac{\sum_{g=1}^{N}l_{k,g}}{N},$$

where $\sum_{g=1}^{N}l_{k,g}$ denotes the number of the nodes that are selected to be activated. We define $f_{c}^{k}$ as the goodness of chromosome *k* and formulate it as:

(10) $$f_{c}^{k}=\alpha_{1}\cdot {\left({ɛ}^{k}\right)}^{\lambda_{1}}-\alpha_{2}\cdot {\left({ℵ}_{t}^{k}\right)}^{\lambda_{2}}.$$

**Figure 6.**
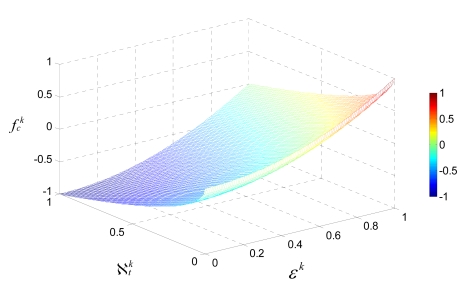
The response surface of $f_{c}^{k}$ in 3-D space, where 0 ≤ *ε^k^ ≤* 1, $0\leq {ℵ}_{t}^{k}\leq 1$, $-1\leq f_{c}^{k}\leq 1$, *α*_1_ = *α*_2_ = 1, **λ_1_** *=* 2 and λ_2_ = 0.5.

Substituting *ɛ^k^* and ${ℵ}_{t}^{k}$ in Equations (8) and (9) into the above equation gives:

(11) $$f_{c}^{k}=\alpha_{1}\cdot {\left(\frac{{\left\|\varpi (k)\right\|}^{2}}{M}\right)}^{\lambda 1}-\alpha_{2}\cdot {\left(\frac{\sum_{g=1}^{N}l_{k,g}}{N}\right)}^{\lambda 2},$$

where *a*_1_ and *a*_2_ are the weighting coefficients, and λ_1_ and λ_2_ are the exponential factors. By doing so, the response surface of $f_{c}^{k}$ has a nonlinear characteristic, as the example shown in Figure 6. The response surface, the represents the distribution of solutions in a 3D space, indicates that the increment of $f_{c}^{k}$ becomes greater as *ε^k^* increases or ${ℵ}_{t}^{k}$ decreases. In this study, we suppose that *a*_1_= *a*_2_=1, *λ =* 2, and *λ*_2_ = 0.5 according to our practical experience after repeated experiments. The constrained boundaries are 0 **≤** *ε^k^* ***≤*** 1, $0\leq {ℵ}_{t}^{k}\leq 1$ and $-1\leq f_{c}^{k}\leq 1$. Thus, the higher value of $f_{c}^{k}$ represents a better solution encoded in chromosome *k*.

#### 3.2.3. Genetic Operations

The GA operations encompass selection, crossover and mutation. There are several kinds of strategies for the selection operation; the most popular ones are the proportional fitness, roulette wheel and tournament. In the CoCMA, we adopt the tournament selection strategy that seeks the optimal fitness of each generation more efficiently. The tournament selection runs a tournament among randomly chosen individuals and then selects the winner for crossover. According to the genetic representation for the CoCMA described above, every allele represents the status of a sensor node: 1-active or 0-inactive. The chromosome with higher fitness implies that the CWSN has a better schedule for sensor nodes to cope with SCP while applying CoCMA. In the process of tournament selection, ten chromosomes are randomly selected from the population, and the elitist chromosome is then selected as parent chromosome 1. The second best chromosome is selected as parent chromosome 2. Both the selected parent chromosomes will breed the new chromosomes of offspring by crossover.

In order to maintain the diversity of a population, determining an appropriate crossover rate is important. The crossover rate *R_c_* specifies the probability of producing two new chromosomes from two selected chromosomes by crossover. For the crossover task, the CoCMA uses a single-point crossover.

Mutation is a genetic operator that helps the entire GA to prevent the population from being trapped in a local optimal solution. It alters one or more values in any possible gene allele. Thus, the new individual is added to the original gene pool. The mutation of GA operations usually occurs after crossover. The mutation rate *R_m_* specifies the probability of altering the value of an allele from its original value. In general, *R_m_* is set to a low value to prevent the search process from turning into a brute-force one. Through the iterative operations of crossover and mutation, the final offspring have a higher fitness on average. According to the paper presented by Eiben *et al.* [33], the crossover rate and mutation rate can be optimized by attaching the parameters to individuals during the evolutionary process. However, we set both of them as fixed values in order to simplify our study. For detailed descriptions regarding the GA, please refer to [35].

#### 3.2.4. Local Search Scheme

In this study, a simple local search strategy is developed to feature the CoCMA with fast convergence and thus further improves the goodness of the population computed by GA operations. For one generation, local search modifies every chromosome (individual) by the proposed local search strategy. This strategy changes the value of every allele from one to zero and determine whether to keep the modification by comparing the fitness of the original and the new chromosomes. The strategy is applied to allele ℓ*_i_,_j_* of chromosome *i*, where*j* = 1, …, *N*. This means that we can get a better schedule for nodes than the original one through the process of local search. The pseudo code of local search scheme used in the CoCMA is described as follows. In this search scheme, let *Pop_k_ =* {*u*_1_, *u*_2_, …, *u_ρ_*} be the population *k*, which is a set of *ρ* chromosomes.

##### Pseudo code of the proposed local search scheme

- **Step 1**: Input a population *Pop_k_ =* { *u*_1_, *u*_2_, *…, u_ρ_* }*_k_* to the local search unit.
- **Step 2**: Let *ui be* the *i*-th chromosome in *Pop_k_*, and *N be* the length of *u_i_*. According to the definition of allele ℓ*_i,j_*, we know that *u_i_* is a binary string composed of ℓ*_i,j_*, where ∀ *i*, 1*≤ j ≤ N*. **For***i* = 1 to *ρ***do**  **For***j =* 1 to *N***do**   **If** the value of ℓ*_i,j_* is equal to one **then**    Record the allele ℓ*_i,j_* in the array *S_i_*.   **End**  **End** **End**
- **Step 3: For***y =* 1 to *ρ***do** Let *h* be the length of *S_y_*.  **For***l* = 1 to *h***do**   *fitness*1 = *fit*(*u_y_*);//*fit*() is the fitness function.   In *u_y_*, let the allele *c_tmp_*, corresponding to the *h* element in *S_y_*, be zero.   *fitness*2 = *fit*(*u_y_*); **//** Re-evaluate the fitness   **If***fitness*1 > *finess*2 **then**    Let *c_tmp_* equal to one.   **End**  **End** **End**
- **Step 4**: Output the improved *Pop_k_*.

The hromosome could be refined to a better one if unnecessary nodes are found. Unlike the conventional GAs, the proposed CoCMA can get better results by performing the local search process. Using the developed local search scheme, the CoCMA converges quickly. It is worthy to note that selecting an appropriate number of chromosomes in each generation is important, since the large number of chromosomes will slow down the process of convergence.

#### 3.2.5. MA Termination

The proposed CoCMA keeps evolving until a termination criterion is satisfied. In the proposed CoCMA, the evolutionary process is terminated when its optimal solution is unchanged for *η* following generations.

### 3.3. Wake-up Scheme

Considering the energy-efficient coverage, we propose a wake-up scheme to awaken some sleeping nodes to recover the uncovered POIs due to energy exhaustions on some sensing nodes. When applying the proposed CoCMA to a CWSN [26], we assumed that the power for the sink of the network is sufficient to carry out the CoCMA operation and the wake-up scheme. In the CWSN [26], each sensor node is capable of communicating with the sink directly. In the following paragraphs, the proposed CWSN-based wake-up scheme will be described in detail.

**Figure 7.**
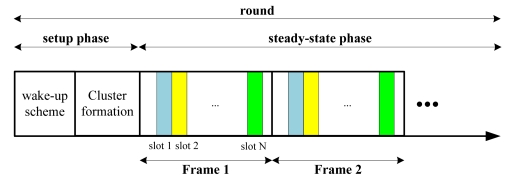
An illustration of time slot arrangement in one round in CWSN.

For the CWSN [26], sensor nodes self-organize themselves into several clusters. In each cluster, only one node can serve as a cluster head. The CWSN uses a TDMA (Time Division Multiple Access) approach to achieve communication between the nodes and their cluster head. Moreover, the cluster head is able to communicate with the remote base station directly and in charge of forwarding the messages received from its cluster nodes to the base station. The head node will set up a TDMA schedule and transmit it to its cluster nodes. The cluster nodes can use the TDMA schedule to arrange the time slots for each node. After the setup phase, the nodes will be in a steady-state phase to forward the data packets to their cluster head. An example of a time slot arrangement in a round is illustrated in Figure 7. In the CWSN [26], the clusters can be re-established in each “round”. A new cluster head is also elected in every round.

When the sink receives the signal transmitted from a dying node, it will determine which nodes should be active and then activates them, when another new round comes. According to the MA-based schedule for sensor nodes, some sensor nodes are switched to sleeping mode, and the remaining sensor nodes are responsible for the sensing task. The MA-based schedule of sensor nodes is conducted in the first round, and the proposed wake-up scheme awakens some sensor nodes in the next round if there is one sensor node about to run out its energy.

According to the coverage vector, which is defined in Section 3.2.2, the sensing coverage can be evaluated by using bitwise Boolean operations. If one node *n_i_* exhausts its energy, the sink will re-evaluate the coverage of the CWSN and find the uncovered POIs by:

(12) $$\pi_{\text{uncovered}}^{n_{i}}=\pi_{n_{i}}^{\Delta }⊕\pi_{n_{i}}^{\nabla },$$

where ⊕ denotes the Boolean exclusive disjunction (also called exclusive or), $\pi_{n_{i}}^{\Delta }$ denotes the original synthetic coverage vector of all sensor nodes including sensor node *n_i_*, $\pi_{n_{i}}^{\nabla }$ denotes the synthetic coverage vector without sensor node *n_i_*, and $\pi_{\text{uncovered}}^{n_{i}}$ denotes the vector that indicates which points are not uncovered. Some definitions should be given before proposing the wake-up scheme.

#### Definition 1

Let node *n_i_*∈*C*, where *C* is the set of all sensor nodes, and *i* ∈ [1, *N*].

#### Definition 2

The *u_i_* denotes the set of all neighboring nodes of *n_i_*, where *i* ∈ [1, *N*]. Here *u_i_* is defined as *u_i_* = {*n_j_* ∈*C*|distance(*n_i_, n_j_*)<*λ*·*r_s_, i*≠*j, j*∈ [1, *N*]}, where *λ* is a constant. We suppose that two nodes are neighbors if the Euclidean distance between them is smaller than *λr_s_*.

#### Definition 3

Let *ψ_i_* be defined as $\psi_{i}=\{n_{*}\in u_{i}|\sum_{\forall h}\pi_{*}\cap \pi_{\text{uncovered}}^{n_{i}}\geq 1\}$, which represents the set of the nodes that have the overlapped POIs between their synthetic coverage vectors and $\pi_{\text{uncovered}}^{n_{i}}$, where π* denotes the synthetic coverage vector of n_*_, and *h* denotes each vector element in $\pi_{*}\cap \pi_{\text{uncovered}}^{n_{i}}$. In addition, let *s_ψi_,_k_* represents the *k*-th possible subset of *ψ_i_*, and *χ* represents the number of subsets of *ψ_i_*.

In order to recover the lost network coverage using sleeping nodes, the wake-up scheme will seek the neighboring nodes of *n_i_* and awaken some of them by the following procedure.

##### Procedure of the wake-up scheme

**variable** best_comb, best_value = 0;map, res = [0, 0, …,0], length = M;**If** node n_i_ has exhausted its energy and can not work correctly **then** **For** k = 1 to χ **do****For** every element d of s_ψi,k_**do**   map = π_d_ ∪ map;  **End**  
$\text{res}=\text{map}\cap \pi_{\text{uncovered}}^{n_{i}};$  **If**$\sum_{\gamma \in \text{res}}\gamma +\frac{1}{\text{size}(s_{\psi ,k})+1}>\text{bes}t\_\text{value}$**then**   
$\text{best}\_\text{value}=\underset{\phi \in \text{res}}{\text{∑}}\phi +\frac{1}{\text{size}(s_{\psi ,k})+1};$   best_comb= s_ψ,k_;  **End** **End** *output = best_comb*;**End**

The main objective of the wake-up scheme is to generate an optimal schedule to awaken some sensor nodes in a sleeping mode. Similar to MAs, the wake-up scheme also emphasizes on energy-efficient coverage. The proposed wake-up scheme requires the sink to transmit a wake-up signal to the neighboring nodes of the dying node from the sink. If these sensor nodes constantly receive the signals, energy consumption will increase significantly. To deal with this problem, some studies [36,37] and corporations [30,31] have proposed a radio wake-up circuit to transmit and receive wake-up signals through different radio channels. In general, transmitting or receiving a packet wastes more energy than performing a sensing task (see Tmote Sky [30] and Mica [31]). In this way, a considerable amount of energy on the sensor nodes can be saved by turning off the onboard communicating module. Once the wake-up circuit receives a wake-up signal, it will activate some of the sensor nodes. The wireless sensor node equipped with wake-up capability has become a commercial standard. As a result, we suppose that each sensor node in the applied CWSN has a built-in wake-up module.

As mentioned above, the sink will awaken some sensor nodes according to the optimal schedule of nodes yielded by the wake-up scheme at the beginning of the next round. If any sensor nodes die again, the wake-up scheme will re-evaluate the coverage and decide to awaken some other sensor nodes to recover the uncovered POIs. Through this way, the coverage preservation of POIs can be achieved effectively.

## 4. Performance Evaluation

In this section, we suppose that a virtual field is monitored by a CWSN with some POIs. The numbers of nodes and POIs are predetermined by the administrator. Each POI and sensor node is stationary after either random deployment or predetermined deployment. All the simulations are conducted on the MATLAB platform.

### 4.1. Convergence of the CoCMA

In order to verify the quick-convergence characteristic of the CoCMA, we evaluate the computing time and the fitness of the CoCMA applied to various sizes of the CWSNs. The results will be compared with those of GAs. We consider 64 POIs randomly distributed in a 100 m × 100 m monitoring area and the number of deployed nodes varies from 50 to 500. The sensing range of a node is fixed as 17.675 m, regardless of the number of deployed sensor nodes. All sensor nodes are randomly distributed. Each experimental simulation was repeated 30 times.

In general, GAs need more generations to generate an optimal solution because of their evolutionary constraints. However, the CoCMA is able to generate the optimal solution within fewer generations as the additional local search scheme is used. We suppose that the CoCMA and GA use the same criterion to stop the evolutionary process. The evolutionary process is terminated when the fitness of the best chromosome is higher than the predetermined threshold. The thresholds of fitness for different network sizes are determined according to our previous experimental studies. We found that the CoCMA has a great leap of fitness around the second generation based on the experimental results. The discrete timestamp and fitness are recorded when one generation finished. Both timestamp and fitness of the GA are also recorded. We utilize a cubic spline interpolation to fit the time-fitness curve for the CoCMA. We then compare the computing time of the CoCMA with that of the GA at different fitness values. The corresponding simulation results are shown in Table 1.

Note that the higher fitness implies a better energy efficiency schedule for nodes according to the definition of fitness function mentioned above. Table 1 clearly indicates that the CoCMA can provide an optimal schedule for nodes that is better than that of GA. We can see that CoCMA takes lesser computing time to yield solutions with better fitness than that in GA. The advantage of CoCMA becomes more apparent when the network size increases. This is because the SCP becomes more complicated as the numbers of the deployed nodes and POIs increases. In the case of network with 500 nodes, the average computing time for the CoCMA at a fitness value of 0.5 is 34.64 second, a 69.3% shorter than that for the GA. In all other cases, the proposed CoCMA is also capable of reaching higher fitness but using lesser time; the average computing time is several times faster than that of the GA. In summary, the operation performance is both convergence and fitness yielded by the CoCMA is more superior to those of the GA, as the nodes deployed in the CWSN become denser.

### 4.2. Network Lifetime Prolongation and Coverage Preservation

In this section, we present the evaluation results for the performance of the CoCMA with respect to network lifetime prolongation and coverage preservation. The CoCMA is applied to the sink and used to control the activation or inactivation of sensor nodes to maintain higher coverage ratio of POIs with energy efficiency. In this scenario, we suppose that there are 64 POIs and 400 nodes distributed uniformly in a sensing field with size of 100 m × 100 m. The initial energy for every node is the same, and all the nodes are assumed homogenous. Similarly, the nodes share the same sensing range of 17.675 meters and can communicate directly with the sink and other nodes in the sensing field. The corresponding parameters of this simulation scenario are summarized in Table 2. Additionally, the nodes are deployed to form a CWSN [26].

**Figure 8.**
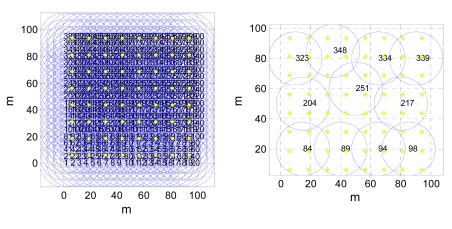
An illustration of uniform deployments of sensor nodes and POIs in the sample network and the simulation result after applying the CoCMA. The yellow star-shaped points represent POIs, and the blue circles represent the sensing ranges for nodes.

For the simulation scenario of uniform deployments, the redundant nodes can be efficiently found by the CoCMA and then be inactivated to conserve energy. Figure 8 (left) shows the initial deployment of nodes and POIs. Based on the proposed MA-based schedule for nodes, the redundant nodes are determined and inactivated. The remaining nodes are still activated and they are responsible to perform sensing tasks. Figure 8 (right) shows the coverage for POIs after applying the MA-based optimal schedule. In this simulation case, only 2.75% of nodes need to be activated to cover all POIs, which are all 1-coverage.

**Figure 9.**
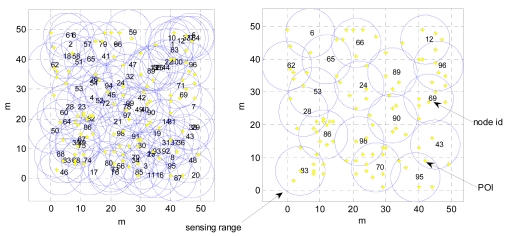
Illustration of random deployments of sensor nodes and POIs in the sample network and the simulation result after applying the CoCMA. (Left) A random deployment of 100 sensor nodes and 100 POIs in a 50 m × 50 m sensing field. (Right) The optimal schedule for nodes is yielded by the CoCMA.

Figure 9 shows the random deployment for a CWSN with POIs. The adopted parameters for the network are summarized in Table 2. In this simulation, there are 100 nodes and 100 POIs randomly deployed in a 50 m × 50 m sensing field, as shown in the left of Figure 9. At this stage, the CoCMA is not applied yet. We note that there are many redundant sensor nodes in this sample network. After applying the CoCMA to the network, the new coverage for POIs is depicted in the right of Figure 9. It is clearly seen from this figure that a better energy-efficient coverage is achieved. The simulation data shows that 72% of the sensor nodes have been switched to a sleeping mode by the assistance of the CoCMA. In this case, only 28% of sensor nodes need to be active. This simulation demonstrates that the given CWSN using the CoCMA is able to monitor all 1-covered POIs and achieves a full coverage.

**Figure 10.**
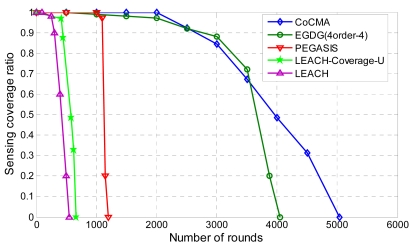
Sensing coverage ratio versus the number of rounds in the uniform deployment scenario.

In the next simulations, we will verify the feasibility of applying the proposed algorithm to a CWSN [26]. The sink node with sufficient power is able to arrange the optimal schedule for every node in the sensing field. When one node exhausts its energy, the wake-up scheme in the CoCMA can determine which neighboring nodes need to be awakened to recover the uncovered POIs. Therefore, the sensing coverage ratio and the number of active nodes are managed simultaneously. To compare the performance of the CoCMA with other four algorithms, simulations were conducted using the same sample network mentioned earlier under uniform deployments of nodes and POIs. The simulation results are compared to those of the Grid-Based Data Gathering Scheme (EGDG) [27], LEACH-Coverage-U [28], LEACH [26] and PEGASIS [29]. Figure 10 depicts the sensing coverage ratio versus the number of rounds. It clearly indicates that PEGASIS, LEACH-Coverage-U and LEACH provide poor capabilities in maintaining the sensing coverage ratio. The proposed CoCMA maintains the sensing coverage ratio at 100% until the 2,000^th^ round which doubles that provided by the PEGASIS. The proposed CoCMA and EGDG yielded similar results in the interval between the 2,000^th^ and the 3,500^th^ round, but the sensing coverage ratio of the EGDG method decreases to around 97% at the 2,000^th^ round. The advantage of the proposed CoCMA becomes significant starting from the 3500^th^ round. Moreover, the network lifetime is prolonged to 5,118 rounds by the CoCMA, which lasts about 1,000 rounds longer compared to the EGDG method.

**Figure 11.**
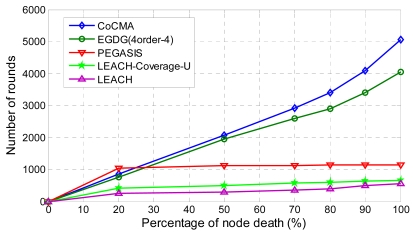
The number of rounds versus percentage of dead nodes (%) for the uniform deployment scenario.

Figure 11 shows the number of rounds versus the percentage of dead nodes. The EGDG, PEGASIS, LEACH-Coverage-U, and LEACH methods lose their 50% of nodes at the 1950^th^, 1120^th^, 502^th^, and 300^th^ round, respectively. The simulation result demonstrates that the proposed CoCMA significantly prolongs the lifetime of the network, even outperforms the EGDG method. We observe that the nodes die rapidly using PEGASIS, LEACH-Coverage-U and LEACH methods, since they do not have node-scheduling strategies. On the contrary, the EGDG and the proposed CoCMA can inactivate the redundant nodes via node-scheduling strategies that save much energy, so the longer network lifetime can be obtained. A longer network lifetime and higher sensing coverage are important for coverage-preserving sensor networks. The simulation results show that the CoCMA is able to achieve these missions. Thereby, the QoS of the network can be improved by using the CoCMA.

**Figure 12.**
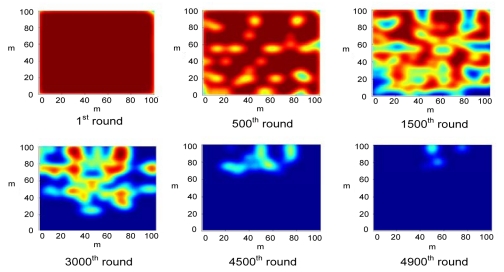
Spatial distribution of the remaining energy for the CWSN during simulation. The dark red regions indicate that the regions reserve more energy, and the dark blue regions preserve less remaining energy.

In order to compute the energy distribution over the network, a 2-D Gaussian distribution is utilized as a point spread function to estimate the spatial strength of energy. Figure 12 depicts the energy distribution over the entire network at different rounds of the simulation. In CWSNs, cluster heads consume most energy since they are responsible for data collection, fusion and packet transmission with a sink. As a result, the energy density on a specific sensing area will decrease rapidly if a large number of nodes congregate at a sub-region of the sensing field as time passes. If the cluster is far away from the sink, the energy level of the cluster will also be lowered. Since the sink is located at (50, 200) (which is far from the sensing field) in this simulation case, the nodes located at the bottom of the given CWSN exhausted their energy rapidly because of the long distance between these nodes and sink. An inspection of Figure 12 indicates that most sensor nodes have died in the sensing field from the sub-figure of the 4,900^th^ round except for the sensor nodes at the top of the sensing field.

**Figure 13.**
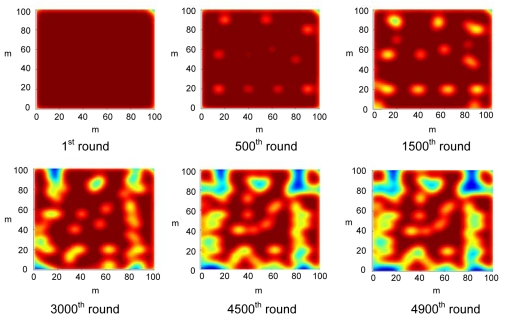
Spatial distribution of the remaining energy for the CWSN, under a same uniform deployment but with previously mentioned case for a uniform deployment, except for a different position of the sink which is changed to (50, 50).

Clearly, the position of a sink will significantly affect the distribution of the remaining energy for the entire CWSN. If the sink is placed at the center of the CWSN, the remaining energy will be distributed over the field evenly, which can be seen in Figure 13. Since the average distance between nodes and sink in Figure 13 is less than that in Figure 12, the total remaining energy in Figure 13 is higher than that in Figure 12. Hence, the network lifetime in Figure 13 is longer than that in Figure 12. In addition, the energy congregated in the surrounding regions of the sink as time passes because the distance between these nodes and the sink is shorter. In Figure 13, some light red regions appear at the 500^th^ round, which implies that some of the active nodes have consumed much energy. At the 1500^th^ round, the original light red regions extended outward, and their centers become yellow-green representing lower remaining energy. After the 1,500^th^ round, we observe the same phenomenon in the extension of light regions. This is because the nearby nodes were awakened to replace the original dying ones. Thus, these awakened sensor nodes will start consuming energy and result in the extension of light regions.

Through the proposed CoCMA, preserving the sensing coverage and prolonging the lifetime can be carried out successfully in the CWSN. In CWSNs applying the CoCMA, the position of the sink will affect the performance of the CWSN according our simulation results. The spatial distribution of energy is more even if the position of the sink approximates the geometric center of the CWSN. This is caused by the innate restriction in CWSNs. We will keep studying the related issues in the future.

## 5. Conclusions

In this paper we have proposed an energy-efficient coverage-preserving algorithm, called CoCMA, which encompasses optimization for initial deployments using a MA and a wake-up scheme. The CoCMA turns off the redundant nodes according to the MA-based schedule for nodes in order to save energy. It also used the wake-up scheme to awaken some sensor nodes in a sleeping mode through an optimization strategy to preserve the sensing coverage of the CWSN. The experimental results show that the proposed CoCMA can be successfully applied to the CWSNs. It minimizes the number of active nodes during the operation period of the CWSN, so the sensing coverage is better preserved comparing with the LEACH, LEACH-Coverage-U, PEGASIS and EGDG. Moreover, the performance of the CoCMA could be significantly improved if the positions of the sink are chosen and the nodes are chosen appropriately. With the promising results demonstrated in this paper, the proposed CoCMA might be applied to other WSNs with different structures, and we reserve this as future work.

## Figures and Tables

**Table 1.** Comparison of average computing time (second) for the CoCMA and GA at different fitness values. The number of nodes varies from 50 to 500. There are 64 positions of POIs randomly located in a CWSN with sensing field of 100 m × 100 m. Every case is repeated 30 times.

**NODE# 50**							**100**		
fitness	GA	Std.	CoCMA	Std.	fitness	GA	Std.	CoCMA	Std.
0.32	3.21	11.45	0.9	0.36	0.53	2	1.7	2.14	0.09
0.3	0.06	0.05	0.82	0.18	0.58	0.58	0.34	2.08	0.09
0.25	0.06	0.05	0.69	0.1	0.45	0.31	0.06	1.98	0.1
**NODE# 200**							**300**		
fitness	GA	Std.	CoCMA	Std.	fitness	GA	Std.	CoCMA	Std.
0.59	21.21	13.31	6.23	0.14	0.55	27.34	13.37	12.99	0.41
0.55	6.62	3.59	6.15	0.14	0.5	6.17	1.5	12.84	0.41
0.5	1.45	0.38	6.04	0.14	0.45	1.58	0.34	12.7	0.41
**NODE# 400**							**500**		
fitness	GA	Std.	CoCMA	Std.	fitness	GA	Std.	CoCMA	Std.
0.52	53.49	29.81	22.43	0.49	0.5	112.8	66.8	34.64	0.78
0.5	24.67	8	22.36	0.49	0.45	11.64	3.74	34.41	0.79
0.45	4.53	1.35	22.16	0.49	0.4	2.1	0.44	34.16	0.79

**Table 2.** Parameters used in the CWSN-type sample network.

a) The sensing range *r_s_* is 17.675 meters. b) *λ* is 1. Nodes with distance less than 17.675 meters are defined as neighbors. c) *η* = 20. a) Crossover rate *R_c_* = 0.5, mutation rate *R_m_* = 0.07. d) *E_elec_* = 50 nJ/bit, *E_amp_* = 100 pJ/bit/m^2^, *E_DA_* = 5 nJ/bit/report, *β* = 2. e) A sink is located at the coordinate (50, 200), which is far from the sensing field and power-rich. f) The size of every data packet is 2,000 bits, i.e., *H* = 2,000. g) Initial energy = 0.25 Joules.
